# Genomic basis and functional characterization of the exopolysaccharide production by a thermotolerant *Bacillus* isolated from Tolhuaca hot spring

**DOI:** 10.3389/fmicb.2025.1622325

**Published:** 2025-08-04

**Authors:** Cynthia Meza, Benjamin Sepulveda, Nicolás Flores-Castañón, Francisca Valenzuela, Catherine Ormeño, Alexis Castillo, Alex Echeverría-Vega, Sura Jasem Mohammed Breig, Saja Mohsen Alardhi, Alex Gonzalez, Bárbara Mora-Lagos, Aparna Banerjee

**Affiliations:** ^1^Doctorado en Biotecnología Traslacional, Facultad de Ciencias Agrarias y Forestales, Universidad Católica del Maule, Talca, Chile; ^2^Ingeniería en Biotecnología, Facultad de Ciencias Agrarias y Forestales, Universidad Católica del Maule, Talca, Chile; ^3^Doctorado en Ciencias Biomédicas, Instituto de Ciencias Biomédicas, Facultad de Ciencias de la Salud, Universidad Autónoma de Chile, Talca, Chile; ^4^Functional Polysaccharides Research Group, Instituto de Ciencias Aplicadas, Facultad de Ingeniería, Universidad Autónoma de Chile, Sede Talca, Talca, Chile; ^5^Centro de Investigación de Estudios Avanzados del Maule (CIEAM), Vicerrectoría de Investigación y Postgrado, Universidad Católica del Maule, Talca, Chile; ^6^Biology Department, Al-Rasheed University College, Baghdad, Iraq; ^7^Nanotechnology and Advanced Materials Research Center, University of Technology, Baghdad, Iraq; ^8^Laboratorio de Microbiología Ambiental y Extremófilos, Departamento de Ciencias Biológicas y Biodiversidad, Universidad de Los Lagos, Osorno, Chile; ^9^Programa de Investigación en Biomedicina y Biotecnología en Salud, Departamento de Salud, Universidad de los Lagos, Osorno, Chile; ^10^Instituto de Ciencias Biomédicas, Facultad de Ciencias de la Salud, Universidad Autónoma de Chile, Temuco, Chile

**Keywords:** *Bacillus*, exopolysaccharide, anticancer activity, emulsifying activity, genomics

## Abstract

*Bacillus licheniformis* Tol1, a thermotolerant bacterial strain isolated from the Tolhuaca hot spring in Chile, was investigated for its genomic features and the functional properties of its exopolysaccharide (EPS). The whole-genome sequencing revealed ∼4.25 Mbp genome with a GC content of 45.9% and a rich repertoire of genes associated with environmental stress adaptation, antibiotic resistance, sporulation, biofilm formation, and EPS biosynthesis, including the presence of epsD and epsC. The strain also harbored intact prophage elements and a Type I-A CRISPR-Cas system, indicating potential horizontal gene transfer and genome plasticity. Confocal microscopy revealed robust biofilm formation at 45–55°C under neutral to slightly alkaline pH, with strong EPS matrix development. EPS production was optimized using OFAT and Response Surface Methodology (RSM), achieving a yield of 2.11 g L^−1^ under optimized conditions, which was further validated using an Artificial Neural Network (ANN) model (*R*^2^ = 0.9909). The EPS exhibited promising antioxidant activity and significant emulsification potential across various vegetable oils, which were comparable or superior to commercial bacterial EPS xanthan gum. Notably, the EPS also showed cytotoxic effects against AGS gastric adenocarcinoma cells, reducing viability by 38.38 and 37% at 50–100 μg μL^−1^ concentrations, respectively, suggesting potential anticancer activity. Altogether, the study highlights *B. licheniformis* Tol1 as a multifunctional thermophile with valuable biotechnological potential, particularly for applications in food, pharmaceutical, and biomedical industries.

## 1 Introduction

Thermophilic microorganisms are a diverse group of extremophiles adapted to thrive in high-temperature environments such as hot springs, fumaroles, geysers, volcanic soils, and deep-sea hydrothermal vents (Mehta and Satyanarayana, 2013). These organisms have evolved unique metabolic pathways and structural adaptations, which make them valuable resources for various biotechnological applications (Persidis, 1998; Mohammadipanah and Wink, 2016). Among thermophiles, species of the genus *Bacillus* are particularly versatile, known for their production of industrially relevant enzymes, antibiotics, and exopolysaccharides (EPSs) (Mishra and Jha, 2013). EPSs are extracellular high-molecular-weight polymers produced by microorganisms, which play vital roles in biofilm formation, stress tolerance, and cell adhesion (Freitas et al., 2011). These biopolymers exhibit a wide range of functional properties, including antioxidant activity, anticancerous effect, and emulsification potential, making them suitable for food, medical, biopharmaceutical, and cosmeceutical applications (Patten and Laws, 2015; Petrova et al., 2021; Salimi and Farrokh, 2023). The stability and functionality of EPSs derived from thermophilic microorganisms under extreme conditions further enhance their industrial relevance (Mohammadipanah and Wink, 2016). The geothermal ecosystems of Chile, including the Tolhuaca hot spring (~38 °S, ~71 °W), are rich reservoirs of microbial diversity. Despite their potential, these ecosystems remain underexplored (Delbarre-Ladrat et al., 2017). Isolation and characterization of thermophilic microorganisms from these environments can lead to the discovery of novel strains with unique metabolic features. For example, EPSs produced by thermophilic *Bacillus* and related genera from similar environments in Chile have shown significant antioxidant and emulsifying activities, suggesting potential industrial applications (Banerjee et al., 2022; Sarkar et al., 2024). Furthermore, thermophilic *Bacillus* species have demonstrated a Generally Recognized As Safe (GRAS) profile due to the absence of virulence factors and the presence of beneficial secondary metabolites, increasing their potential for applications in food and biomedical industries (Harirchi et al., 2022).

Genomic studies have provided valuable insights into the metabolic pathways and regulatory mechanisms involved in thermophilic behavior and EPS biosynthesis. The whole-genome sequencing of EPS-producing thermophilic bacteria has revealed genes linked to stress tolerance, polysaccharide biosynthesis, and nutrient utilization (Freitas et al., 2011; Mohammadipanah and Wink, 2016). For instance, the genomic analysis of *Geobacillus* sp. WSUCF1 and *Brevibacillus thermoruber* 423 demonstrated pathways critical for EPS production and adaptation to high temperatures (Petrova et al., 2021). The genome-based approach has been previously evaluated to study the metabolic potentials and EPS production of *Bacillus paralicheniformis* CamBx3 isolated from a Chilean hot spring named Campanario, where the EPS demonstrated the property of a thermoresistant, antioxidant, and viscoelastic gel, optimum for use as additive in food processing (Narsing Rao et al., 2024). Moreover, some microbial EPSs have demonstrated promising anticancer potential, capable of reducing tumor cell viability through mechanisms like apoptosis and cell cycle arrest (Xiao et al., 2020; Abdelnasser and Abu-Shahba, 2024). However, integrative genomic and functional characterizations of EPS-producing thermophiles from Chilean geothermal niches remain scarce. In this study, we focus on *Bacillus licheniformis* Tol1, a thermotolerant strain isolated from the Tolhuaca hot spring in Chile. This strain exhibits optimal growth at 55°C and produces EPS with notable antioxidant and emulsifying properties. Through whole-genome sequencing and annotation, we aim to elucidate the genetic determinants underlying its thermotolerant, extreme lifestyle, adaptations under extreme environments, EPS production, and the biological activities of the EPS. Understanding these genetic factors is essential for harnessing the biotechnological potential of *B. licheniformis* Tol1 in various industrial applications, including food preservation, pharmaceuticals, and environmental remediation. This research highlights the untapped potential of geothermal ecosystems in Chile as sources of microorganisms with industrially relevant characteristics. By combining genomic and functional analyses, we provide a comprehensive characterization of *B. licheniformis* Tol1, paving the way for its integrative application in biotechnology.

## 2 Materials and methods

### 2.1 Study site and sample collection

The samples were collected from the hot springs located at the Tolhuaca thermal area (38°13′59.09”S, 71°43′20.3”W). Situated in the pre-Andean mountain range at 1,150 m.a.s.l., and ~33 km from the city of Curacautín (Araucanía Region of Chile). This geothermal region is characterized by high volcanic activity, influenced by nearby volcanoes such as Tolhuaca, Lonquimay, Llaima, and Sierra Nevada. In the study zone, a 500 mL thermal water at ~96°C was collected aseptically into sterile containers and transported to the laboratory under controlled conditions for further processing.

### 2.2 Isolation and polyphasic characterisation

The bacterial strain Tol1 was isolated from the water samples described above. The samples were subjected to a 10-fold serial dilution in sterile water. Aliquots (50 μL) from each dilution were spread on petri dishes containing 23 g L^−1^ of Nutrient Agar (Difco™) and incubated at 55°C for 24–48 h. Distinct mucoidal isolated colony was picked and reisolated by successive streaking on fresh agar plates until pure cultures were obtained, showing a shiny, mucoid appearance due to EPS production.

### 2.3 Taxonomic identification of EPS producing thermotolerant by whole genome analysis

Genomic DNA from strain Tol1 was isolated from freshly grown bacteria (~0.5 g) in NA plates using DNA power soil kit (Sigma Aldrich) according to the manufacturer’s instructions. Although the culture was purified, we used the DNA PowerSoil Kit (Sigma-Aldrich) as it has been demonstrated to efficiently lyse robust Gram-positive bacterial cells, such as *Bacillus* species, which can be difficult to lyse using standard kits due to their thick peptidoglycan-rich cell walls. This choice ensured high-quality and high-yield DNA (396.3 ng μL^−1^, A260/A280 = 1.87) suitable for downstream genome sequencing. DNA concentration and quality were assessed using NanoDrop 1000 (Thermo Fisher Scientific) and Qubit 4.0 (Thermo Fisher Scientific). The genome sequencing was performed using Illumina Novaseq PE150 (Illumina, Inc., San Diego, CA, USA) platform using paired-end sequencing strategy. Raw reads were filtered, and high-quality paired-end reads were assembled using SPAdes (v3.15.5) (Bankevich et al., 2012). CheckM was used to evaluate strain Tol1 genome quality (Parks et al., 2015). The identification of rRNA genes was carried out using RNAmmer (version 1.2) (Lagesen et al., 2007). The taxonomic identification of Tol1 was carried out using the 16S rRNA sequence as a phylogenetic marker, obtained through the barrnap software (Galaxy Version 1.2.2) from the sequence of the complete genome and compared using the EzBioCloud server (Yoon et al., 2017) and NCBI BLAST-n. The genome was visualized using Proksee (Grant et al., 2023) and compared with the closest species using BLAST (Altschul et al., 1990). Prodigal was used to identify open reading frames (Hyatt et al., 2010). The genome sequence was submitted to GenBank under the accession number JBNNEF000000000.

*B. licheniformis* Tol1 genome was categorized in a subsystem using an open-source prokaryotic genome annotation system, Rapid Annotation System Technology (RAST) pipeline, compared with the entire available dataset (Aziz et al., 2008). The functionally annotated genes present within the genome were visualized using SEED Viewer. PHAge Search Tool Enhanced Release (PHASTEST) web-based server was used to identify genes related to prophage with the gnome of Tol1 (Wishart et al., 2023). To predict genomic islands, IslandViewer4 was used (Bertelli et al., 2017).

### 2.4 Formation of biofilm by isolated thermotolerant *B. licheniformis* Tol1

The biofilm formation experiment was performed in 50 mL centrifuge tubes using 20 mL of growth medium and 200 μL of inoculum. Over a period of days, we observed the growth of bacteria and the production of EPS from strain Tol1 using confocal microscope (Leica Stellaris 5, Wetzlar, Germany). The sample was stained with DAPI (4′,6-diamidino−2-phenylindole, a fluorescent stain used to label DNA which fluoresces at 358/461 nm) to visualize the bacterial cells, and SYPRO™ Ruby (highly sensitive, detecting glycoproteins with a fluorescence spectrum of 450/610 nm) to visualize the biofilm matrix.

### 2.5 OFAT optimization of EPS production

A bacteriological analysis was performed to determine the optimal culture conditions that maximize EPS production. A one-factor-at-a-time (OFAT) assay was performed under different conditions where EPS production can be visualized by viscosity. The strain was inoculated into LB Agar medium supplemented with different carbon sources (galactose, mannose, glucose, and sucrose), nitrogen sources (yeast extract, peptone, meat extract, and ammonium chloride), different initial pH (5, 6, 7, and 8), temperature (50, 55, 60, 65, 70°C), and finally salinity (NaCl) (2, 5, 7, and 10%). Each experiment was performed in triplicate to reduce experimental error. To precipitate the EPS, bacterial cells in the stationary growth phase were harvested, followed by treatment with 4% trichloroacetic acid (TCA) (w/v) for 30 min (Sarkar et al., 2024). Centrifugation at 4°C, 5000 × g for 20 min was applied to the TCA-precipitated cells to achieve protein precipitation. Then, the chilled, cell-free supernatant was left overnight at 4°C after adding an equal volume of acetone. The next day, centrifugation at 12,000 × g for 20 min was applied to the solvent-coagulated EPS. The acetone precipitation step was repeated with water-dissolved EPS to remove residual TCA and proteins. Pure EPS powder was obtained after dialysis and lyophilization of purified EPS.

### 2.6 RSM optimization

In this study, RSM based on central composite design (CCD) was used to optimize three parameters (sucrose concentration %, NH_4_Cl %, and NaCl %) with five levels of each parameter coded as (−2, −1, 0, +1, +2) were used as independent parameters and EPS production as response. Table 1 presents the coded and uncoded values of independent parameters, coded according to the following Equation 1:

(1) $$\mathrm{xi}=(X_{i}-X_{o}/\mathrm{\Delta X})$$

**Table 1:** Independent parameters and levels in CCD.

Parameter	Unit	−2	−1	0	1	2
Sucrose	%	1.32	2	3	4	4.681
NH_4_Cl	%	1.32	2	3	4	4.681
NaCl	%	2.32	3	4	5	5.681

Where: *xi*: the coded value of the parameter, X_i_: the actual value of the parameter, X_o_: the actual value of X_i_ at center point, ΔX: step change.

The CCD matrix was generated by Design Expert software (version 7) consisting of twenty runs with five replicates of center point (Supplementary Table 1). The experiment was conducted in 200 mL glass flasks with 100 mL of medium. Flasks were prepared and inoculated in triplicate to ensure adequacy in all experiments. The inoculated flasks were incubated at 55°C and 150 rpm for 3 days in a rotary shaker. EPS production was achieved, and the data were analyzed in design expert software to evaluate the effects of each parameter and their interaction to predict the production of EPS as described in the quadratic optimization model (Equation 2):

(2) $$Y=\beta_{0}+\sum_{i=1}^{k}\beta_{0}x_{i}+\sum_{i=1}^{k}\beta_{\mathrm{ii}}x_{i}^{2}+\sum_{i=1}^{k}\sum_{i=2}^{k}\beta_{\mathrm{ij}}x_{i}x_{j}$$

Where Y is the predicted response, β_0_ is the intercept term, β_i_ is the linear effect, β_ii_ is the squared effect, β_ij_ is the interaction effect, and X_i_ and X_j_ are input variables that influence the response variable Y.

Design expert software is a beneficial tool utilized for designing experiments and data analysis of their responses (Suryawanshi et al., 2019). The optimization model was statistically evaluated to assess analysis of variance (Table 2). Model fitness expressed by R^2^, R _adjust_, R _predict_, adequate precision, standard deviation, coefficient of variation, mean, press, F- value, P > F prob. and lack of fit, other statistical terms used for statistical analysis (Breig and Luti, 2021; Sarkar et al., 2024). Depending on the regression model, an optimization plot can be developed by using Design expert software to identify the optimum concentrations [sucrose concentration %, NH_4_Cl %, and NaCl %] that maximize EPS production, briefly, all independent parameters selected as in range mode except the initial concentration of dye selected as maximum mode and response EPS production as maximize to construct ramp chart for optimization description. The optimum parameters obtained were validated in the laboratory in order to verify the predicted EPS production.

**Table 2:** Analysis of variance for EPS production by *B. licheniformis* Tol1.

Source	Sum of squares	Df	Mean square	F-value	*p*-value Prob > F
Model	5.58865394	9	0.620961549	15.87727	< 0.0001***
A-Sucrose	0.2273417	1	0.2273417	5.812863	0.0366***
B-NH_4_Cl	0.33890262	1	0.33890262	8.665346	0.0147***
C-NaCl	5.0906E-05	1	5.09059E-05	0.001302	0.9719**
AB	0.03062812	1	0.030628125	0.783126	0.3970**
AC	0.00112812	1	0.001128125	0.028845	0.8685**
BC	0.41632813	1	0.416328125	10.64503	0.0085***
A^2^	2.46169661	1	2.461696612	62.94272	< 0.0001***
B^2^	0.99042948	1	0.990429482	25.32413	0.0005***
C^2^	1.97280022	1	1.972800215	50.44221	< 0.0001***
Residual	0.39110106	10	0.039110106		
Lack of Fit	0.14210106	5	0.028420212	0.570687	0.7234
Pure Error	0.249	5	0.0498		
Cor Total	5.979755	19			
R^2^	0.934596				
Adj R^2^	0.875732				
Pred R^2^	0.757916				
Adeq Precision	11.67012				
Std. Dev.	0.19776275				
Mean	1.3535				
C. V. %	14.6112118				
PRESS	1.44760231				

Where ** = not significant, *** = significant, sum of square = Total sum of square of selected factors, Df = Degree of freedom, Mean square = Estimate of midel variance, F value = Test for comparing model variance with residual variance, *p*-value Prob > F = Probability of observed *F* value if the null hypothesis is true, Cor Total = Corrected Total Sum of Squares, Adj R^2^ = Adjusted R^2^, Pred R^2^ = Predicted R^2^, Adeq Precision = Adequate Precision, Std Dev. = Standard Deviation, C. V. = Coefficient of variance, PRESS = Predicted Residual Sum of Squares (Breig and Luti, 2021).

### 2.7 Artificial neural networks prediction

Modeling is a useful technique for successfully and economically determining ideal operating conditions and forecasting process results. The normal method for modeling a traditional chemical reactor is to use standard mathematical tools to solve the mass, energy, and momentum balance equations concurrently. Numerous articles document the wide exploration of the use of Artificial Neural Networks (ANN) for process modeling in a variety of scientific and engineering fields (Breig and Luti, 2021; Mahdi et al., 2021; Alardhi et al., 2022; Banerjee et al., 2022; Alardhi et al., 2024). Similar to biological neural networks, artificial neural networks are dynamic, non-linear systems made up of numerous processing units (neurons) coupled to one another. Feedforward neural networks and recurrent neural networks are the two primary categories of ANN. In a feedforward neural network, input data only moves from the input nodes to the output nodes in a forward manner (Al-Jadir et al., 2023; Dawood Salman et al., 2023). Every node in a layer is linked to nodes in the layers above and below it, resulting in a unidirectional flow devoid of backward connections (Sadeghzadeh Ahari et al., 2024). The Multilayer Perceptron (MLP), the most common type of feedforward neural network, usually consists of one or more hidden layers, an input layer, and an output layer. The amount of input variables and the number of variables that need to be predicted correlate with the number of nodes in the input and output layers, respectively (Ayed et al., 2022). An iterative process of trial and error is frequently required to determine the best configuration for the hidden layers, their nodes, and the transfer functions between levels (Annadurai and Lee, 2007). By using an iterative process, several types of networks can be explored with the ultimate goal of finding a structure with fewer layers and nodes that reduce prediction errors (Alardhi et al., 2023b). In this work, the reliability of the ANN models was tested using several indices in addition to the expected and actual results. These markers are MSE and RE, as can be seen in the following Equations 3, 4 (Alardhi et al., 2023a).

(3) $$\mathrm{MSE}=\frac{1}{n}\;\sum_{i=1}^{n}(D_{a}(t)-D_{f}(t))$$

(4) $$\mathrm{RE}=\frac{D_{a}(t)-D_{f}(t)}{D_{a}(t)}\times 100$$

Where: $D_{a}(t)$ and $D_{f}(t)$ are the actual and predicted values at time t.

ANNs were utilized to model the EPS production using MATLAB R2021a software. The neural network was trained by the experimental data set, which consisted of 80 data points and was gathered at the lab scale to estimate the EPS production percent. Several factors of bacterial growth, such as % sucrose (1.32, 2, 3, 4, 4.68) as carbon source, % NH_4_Cl (1.32, 2, 3, 4, 4.68) as nitrogen source, % NaCl (2, 2.32, 3, 4, 5 and 5.68) as inputs, were initially included in the experimental study before being included in the modeling.

**Figure 1:**
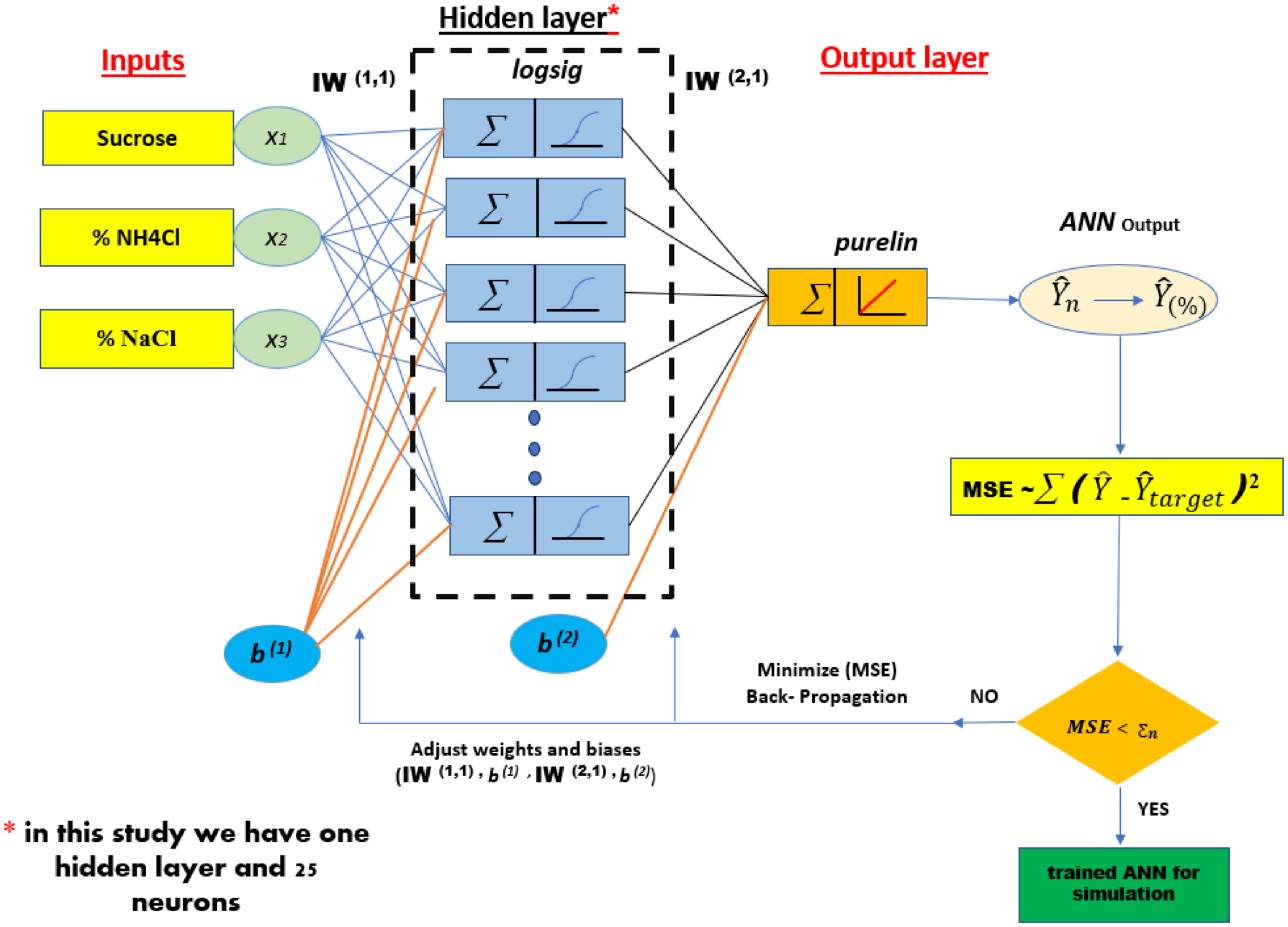
The architecture of the ANN model for prediction of EPS production.

An MLP is composed of an input layer, an output layer, and one or more hidden layers, as illustrated in Figure 1. Each layer is composed of a specific quantity of neurons; the number of neurons in the input and output layers correspond to the number of input and output variables, respectively. The values of neurons in both the hidden and output layers are contingent upon the weights assessed throughout the training phase. A threshold signal is assigned to a weight and incorporated into each neuron within the hidden and output layers. The construction of ANN models involves three stages: training, validation, and testing. Consequently, the data set is divided into three categories: training, validation, and testing. The majority of the data resides in the training dataset, often comprising 70% of the total data. The residual data is allocated between the validation and testing data sets (15 and 15%) respectively.

### 2.8 Functional property elucidation of the EPS

#### 2.8.1 *In vitro* antioxidant activity determination

The radical scavenging activity (RSA) of 2,2-diphenyl−1-picrylhydrazyl (DPPH) was determined following methods adapted from (Shimada et al., 1992). In summary, different concentrations of 200 μL of EPS aqueous solution (0.1, 1, 2, 3, 4 and 5 mg mL^−1^) were mixed with 20 μL (0.2 mM) ethanolic DPPH solution. The mixture was then kept in the dark at 30°C for 1 h and subsequently centrifuged at 5000 × g for 10 min. The absorbance of the supernatant was measured at 517 nm using a microplate reader (Allsheng, Flex A-200, China). The following Equation 5 was used to calculate the DPPH scavenging activity:

(5) [(A−B)/A]×100

Where A is the absorbance of the control supernatant, and B is the absorbance of the sample, respectively.

#### 2.8.2 Anticancer activity

AGS cells, a human Caucasian gastric adenocarcinoma cell line (Cat# 8,909,040), were used in this study. The AGS cell line was acquired from the European Collection of Authenticated Cell Cultures (ECACC) and distributed by Sigma-Aldrich Corporation-ECACC, Oceania Inventory. AGS cells were cultured in RPMI-1640 medium (ThermoFisher, USA) supplemented with 10% (v/v) fetal bovine serum (ThermoFisher, USA) and 1% (v/v) penicillin–streptomycin solution (ThermoFisher, USA). The cells were maintained at 37°C in a 95% humidified atmosphere with 5% CO₂ and subcultured upon reaching 80% confluence. Harvesting was performed following treatment with 0.25% trypsin and 0.02% EDTA (ThermoFisher, USA).

Effects of EPS produced by *B. licheniformis* Tol1 on the cytotoxicity of AGS cells were determined by MTT viability assay. Briefly, 4 × 10^3^ AGS cells were seeded into 96-well plates with 100 μL of culture medium and incubated for 24 h to allow attachment, achieving approximately 50% confluence. Cells were then exposed to various concentrations of each EPS (0.1, 1, 10, 20, 50, and 100 μg μL^−1^) for 72 h. Untreated cells served as controls. Following each incubation period, the culture medium was removed, and the cells were washed with 100 μL of DPBS/Modified (Corning, USA). Subsequently, the cells were treated with MTT (Merck Group, Germany) at a concentration of 0.5 mg/mL and incubated for 2 h at 37°C. Through functional mitochondrial dehydrogenase enzymes, viable cells reduce MTT to form formazan, evidenced of the formation of a purple precipitate. The formazan was then dissolved entirely in 100 μL of propanol. Absorbance was measured at a wavelength of 570 nm using the Infinite NanoQuant spectrophotometer (TECAN, Switzerland). All experiments were conducted in biological and technical triplicates for each condition. Data were analyzed using GraphPad Prism version 10.0.3 software (GraphPad, USA). Cell viability data were evaluated using the Kruskal-Wallis test, followed by Dunn’s *post hoc* test.

#### 2.8.3 Analyses of emulsifying activity

The emulsifying activity of EPS produced by *B. licheniformis* Tol1 was performed using the method described by (Cooper and Goldenberg, 1987), with some adjustments. To assess the emulsifying activity, edible oils were utilized, including sesame, walnut, canola, olive, corn, grape seed, vegetable, and rice oils. The emulsifying property of the EPS was compared with commercially available bacterial polysaccharide xanthan gum (Sigma, St. Louis, Missouri, USA). For this experiment, 2 mL of an aqueous solution of our studied EPS (1 mg mL^−1^) was added, followed by 3 mL of each vegetable oil (2:3 v/v). The mixture was vigorously stirred for 2 min. The oil, emulsion, and aqueous layers were measured at 24-h intervals to evaluate emulsion stability. All experiments were performed in triplicate. The emulsion droplets were measured according to Song et al. (2019). The emulsification index (E_24_) was calculated using the following Equation 6:

(6) $$\left[(\text{volume of the emulsion layer}\times \text{total volum}e^{-1})\times 100\right]$$

## 3 Results

### 3.1 Taxonomic identification of EPS producing thermotolerant *Bacillus licheniformis* Tol1 by whole genome analysis

The genome of *Bacillus licheniformis* Tol1 identified through 16 s rRNA sequence analysis, as annotated through the RAST subsystem, exhibits a comprehensive set of features indicative of its genetic complexity and adaptability. The genome has a total size of 4, 247, 584 base pairs with a GC content of 45.9%, reflecting a balanced composition conducive to thermophilic adaptation (Hu et al., 2022). The assembly metrics highlight a high-quality genome, with an N50 of 2,295,028 and an L50 of 1, indicating that the genome is represented mainly in a single, contiguous segment. The analysis identified 56 contigs containing protein-encoding genes (PEGs), with a total of 4,684 coding sequences and 90 RNA genes, including essential components for transcriptional and translational regulation. From BLAST-n, the highest degree of similarity was found with *Bacillus* sp. (in: Bacteria) strain *licheniformis* 16S ribosomal RNA gene, partial sequence (sequence ID: MH900194.1) with 100% identity percentage. The 16S rRNA sequence of Tol1 was also identified from EzBioCloud server, where the sequence showed highest degree of similarity with *B. licheniformis* type strain ATCC 14580 having 99.92% similarity.

**Figure 2:**
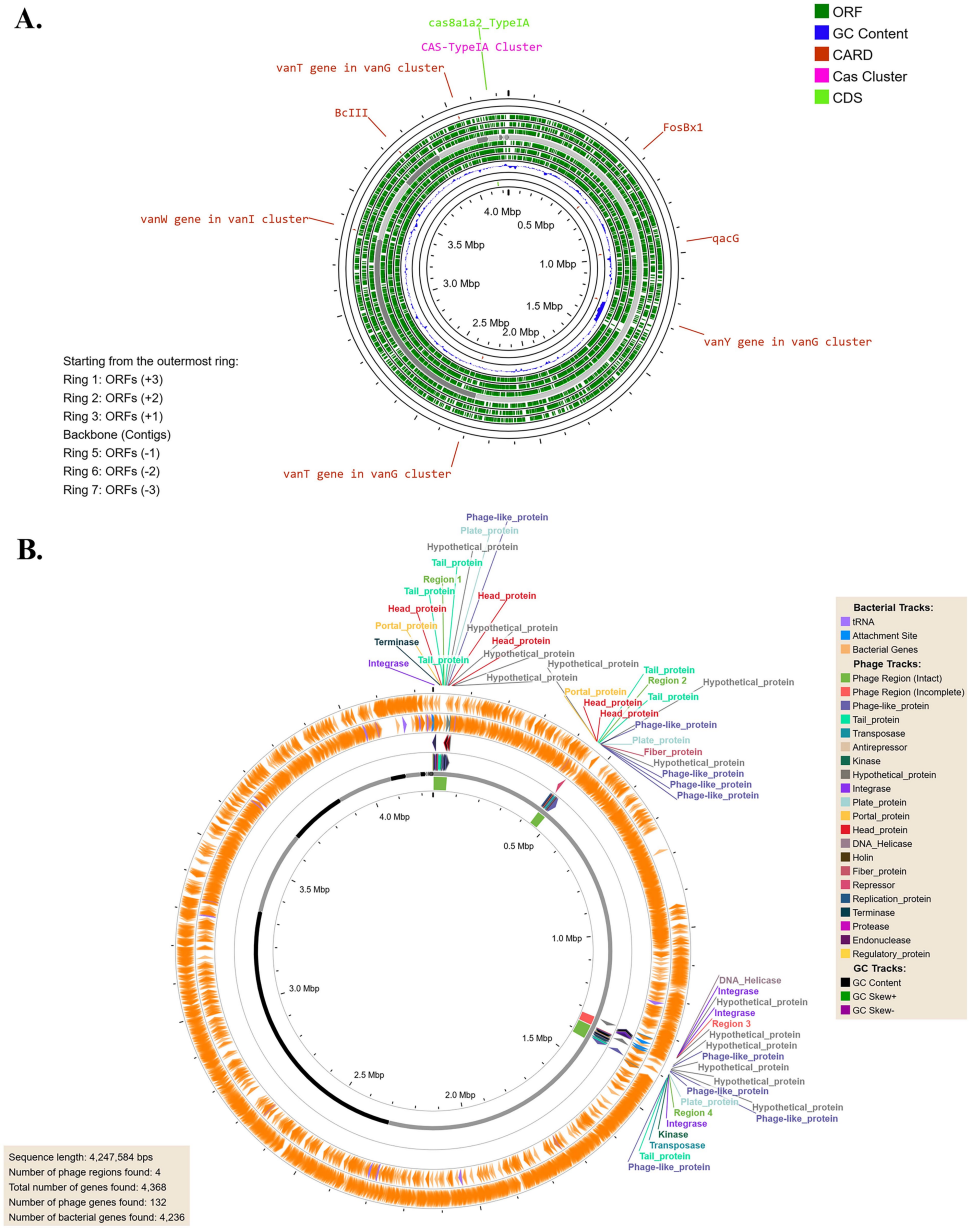
**(A)** Circular genome annotation showing ORFs on both strands, with prominent antibiotic resistance genes (vanT, vanW, vanY, BcIII, qacG, FosBx1) and a type I-A CRISPR-Cas system. **(B)** Identification of prophage regions.

The circular genome annotation of *B. licheniformis* Tol1 (Figure 2A) reveals a metabolically versatile and functionally rich genome, with multiple open reading frames (ORFs) identified across both forward and reverse strands. Notably, the genome contains critical antibiotic resistance genes, including vancomycin resistance clusters (*vanT*, *vanW*, *vanY*) within the *vanG* and *vanI* operons, a *β*-lactamase gene (*BcIII*), and *qacG*, associated with resistance to quaternary ammonium compounds. Additionally, the presence of *FosBx1* indicates resistance to fosfomycin, highlighting the strain’s potential multi-drug resistance. A Type I-A CRISPR-Cas system (*cas8a1a2_TypeIA*) was also identified, suggesting adaptive immunity against foreign genetic elements like plasmids and bacteriophages. The GC content and skew analysis point to regions with functional significance and potential horizontal gene transfer. Collectively, these features underscore the bacterium’s resilience and adaptability to extreme environments while also providing insights into its biotechnological potential and implications for antimicrobial resistance. The genome of *B. licheniformis* Tol1 harbors four prophage regions (Figure 2B), three of which are intact and one incomplete, showcasing its dynamic evolutionary history. The intact regions include a 51.5 Kb prophage related to *PHAGE_Bacillus_phi105* (GC content 40.10%) and a 54.4 Kb prophage associated with *PHAGE_Bacillus_SPbeta* (GC content 36.05%), both of which are known temperate phages commonly found in *Bacillus* species. These intact prophages are fully capable of encoding functional phage particles, suggesting their potential inducibility under stress conditions, contributing to horizontal gene transfer and genome plasticity. The 32.9 Kb intact prophage related to *PHAGE_Brevibacterium_Jimmer2* (GC content 47.59%) highlights possible interspecies genetic exchange, further enhancing the bacterium’s adaptability. The 34.1 Kb incomplete prophage, a remnant of *PHAGE_Bacillus_SPbeta* (GC content 33.82%), may still encode functional genetic fragments that influence host fitness. The diversity of these prophages and their associated genes underscores their potential roles in conferring beneficial traits, such as antibiotic resistance, stress tolerance, or novel metabolic capabilities, aiding *B. licheniformis* Tol1 surviving within the extreme conditions of a hot spring.

The genome of *B. licheniformis* Tol1, annotated using the RAST subsystem, reveals a diverse array of functional features that underscore its metabolic versatility, stress resilience, and potential for biotechnological applications. Among the mapped subsystems, carbohydrate metabolism (314 genes) and amino acid biosynthesis and derivatives (312 genes) dominate, highlighting the bacterium’s capacity to utilize various energy and nutrient sources vital for survival in the extreme conditions of the Tolhuaca hot spring. Dormancy and sporulation pathways (95 genes) are detected, including spore core dehydration, sporulation clusters, biofilm matrix protein TasA, and spore germination genes, all of which contribute to its ability to withstand thermal, osmotic, and nutrient stress. A strong oxidative stress response (17 genes), including protection from reactive oxygen species and glutathione metabolism, coupled with osmoregulation and choline/betaine biosynthesis (11 and 10 genes, respectively), further ensures the organism’s resilience in challenging environments. Interestingly, it has genes for EPS biosynthesis, including the tyrosine-protein kinase EpsD and its modulator EpsC, which are essential for biofilm formation and extracellular polymer production. Stress-specific responses include genes for sigmaB regulation (6), carbon starvation (2), and detoxification (4), providing mechanisms to mitigate cellular damage and sustain metabolic activity during environmental fluctuations. In addition to stress response, *B. licheniformis* Tol1 encodes 20 genes associated with antibiotic and toxic compound resistance, such as fluoroquinolones, beta-lactams, and heavy metals like cobalt, cadmium, and copper, indicating its adaptability and potential for bioremediation. The genome also reveals a robust system for DNA repair (47 genes), including RecA-mediated homologous recombination, UvrABC excision repair, and pathways for non-homologous end-joining and the RecBCD and RecFOR systems. Phage-related genes (16), including those for replication, capsid formation, and packaging machinery, highlight horizontal gene transfer as a driver of genomic plasticity and evolutionary adaptability. Lastly, genes associated with plant hormone biosynthesis (4 auxin biosynthesis genes) suggest its role as a potential plant growth-promoting bacterium. At the same time, no virulence factors were identified, emphasizing its safety for industrial and agricultural applications. Collectively, these features underline *B. licheniformis* Tol1’s potential for sustainable biotechnological processes, from biopolymer production to environmental resilience and agricultural enhancement.

### 3.2 Formation of biofilm by isolated thermotolerant *B. licheniformis* Tol1

**Figure 3:**
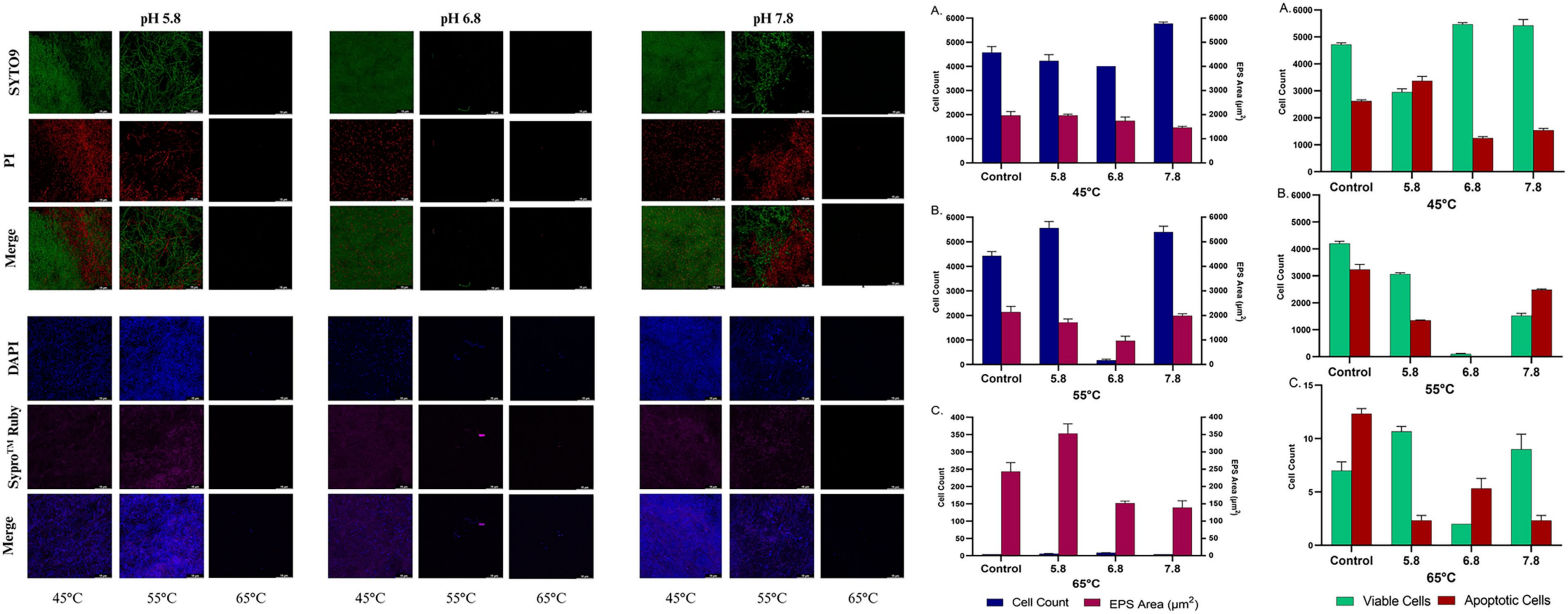
Biofilm formation by *B. licheniformis* Tol1 under different pH and temperature conditions (left panel). On the right, confocal microscopy-based analysis of viable and apoptotic cell populations along with EPS matrix area under incubation temperatures: 45°C **(A)**, 55°C **(B)**, and 65°C **(C)**, and pH conditions (5.8, 6.8, and 7.8). Data represents SD from triplicate experiments.

The biofilm-forming ability of the bacteria *B. licheniformis* Tol1 under varying pH conditions (5.8, 6.8, 7.8) and incubation temperatures (45°C, 55°C, and 65°C) were evaluated using confocal microscopy. SYTO9/Propidium Iodide staining was employed to assess cell viability within the biofilm, while DAPI/SYPRO Ruby staining was used to visualize and quantify (Figure 3) the extracellular polymeric substance (EPS) matrix. At 45°C, *B. licheniformis* Tol1 formed robust biofilms with high viability. Across all pH conditions, viable cells outnumbered apoptotic ones, particularly at pH 6.8 and 7.8, where viable cell counts exceeded 5,000, suggesting favorable biofilm formation conditions. EPS production was also high at this temperature, with the EPS matrix area ranging from ~1,500 to 2,200 μm^2^, supporting the visual observation of dense, well-structured biofilm mats. Further, at 55°C, the strain maintained strong biofilm formation at pH 5.8 and 7.8, with cell counts above 4,000 and EPS matrix areas similar to those at 45°C. However, at pH 6.8, both viable cell numbers and EPS production decreased sharply, suggesting reduced biofilm viability at this specific pH-temperature combination. This indicates a shift in the optimal biofilm-forming window at elevated temperatures. Lastly, at 65°C, biofilm formation was minimal across all pH values. Cell viability drastically declined, with total viable cell counts falling below 15 and EPS matrix areas dropping to negligible levels. These results confirm that 65°C approaches the upper physiological limit for active biofilm development in *B. licheniformis* Tol1, although sparse biofilm fragments were still detectable at pH 7.8 (Figure 3). Overall, the results demonstrate that thermophilic *B. licheniformis* Tol1 forms highly structured and viable biofilms at moderate thermal conditions, particularly at 45–55°C and neutral to slightly alkaline pH. The presence of an extensive EPS matrix further confirms active biofilm development, which supports the bacterium’s resilience in thermal environments and strengthens its biotechnological relevance for thermophilic applications.

### 3.3 OFAT optimization of EPS production

Considering the high apparent viscosity and the ropy behavior of *B. licheniformis* Tol1 in nutrient supplemented LB medium (Supplementary Table 1), the best-optimized carbon source was sucrose, and the nitrogen sources was NH_4_Cl along with NaCl, resulting in an initial EPS production of 0.2 g L^−1^ under pre-optimized condition. This was used for subsequent response surface methodology (RSM) analysis to optimize EPS production statistically.

### 3.4 RSM optimization

RSM based on a central composite design technique has been successfully applied to optimize the possible combination of parameters (sucrose concentration %, NH_4_Cl %, and NaCl %) that maximize EPS production by *B. licheniformis* Tol1 (a maximum of 2.11 g L^−1^ at sucrose concentration 3%, NH_4_Cl 3%, and NaCl 4%) as shown in Supplementary Table 2, the upper and lower limits of each parameter were specified according to initial laboratory study based on previous published research.

From the ANOVA (Table 2) for the quadratic optimization model, the model showed high significance with *F*-value 15.877 (*p*-value >F was <<0.0001); in addition, the model was evaluated by determination coefficient R^2^ = % 93.4, which means the model does not explain %6.6 of total experiments, R^2^ adjust, R^2^ predict for EPS production were 0.875 and 0.757, respectively, which indicated good results with difference less than 0.2. The measurement of the signal-to-noise model was assessed by adequate precision and accepted with a value of more than 4 in this model (11.67). From ANOVA Table 2 for EPS production, it can be noticed most terms exhibited significant effect (*p* < 0.0001). This study evaluated the significance parameters by *p*-values, *p*-values more than 0.1, and revealed that the term was insignificant.

Furthermore, a regression optimization model was constructed, which is a relationship between independent parameters and dependent parameters (EPS production) developed after ANOVA and the regression coefficient determined. The CCD design matrix was fitted with a quadratic optimization model for EPS production in coded parameters as described in Equation 7.

(7) $$\begin{array}{l} \mathrm{EPS}=2.0673+0.1290\times A+0.15752\times B-0.0019\times C-0.0618\\ \times A\times C-0.228\times B\times C-0.4133A^{2}-0.26{\mathrm{2B}}^{2}-0.3699C^{2} \end{array}$$

**Figure 4:**
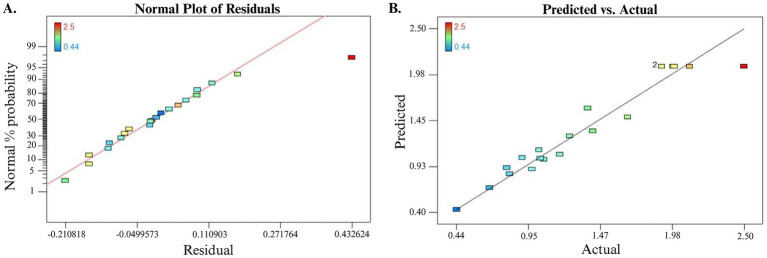
**(A)** Normal plot of residual for EPS production. **(B)** Predicted versus actual plot for EPS production.

In addition to the analysis of variance (ANOVA) and correlation optimization model, regression analysis can be applied to evaluate the best-fit line among the experiments, which can be visualized by a normal plot of residual (Figure 4A). Moreover, as shown in Figure 4B, the observed experimental values against predicted output values for EPS production exhibited good aggregation between observed values and predictive values estimated by the quadratic optimization model. Hence, the developed optimization model can be generalized for EPS production by *B. licheniformis* Tol1 under the same condition.

**Figure 5:**
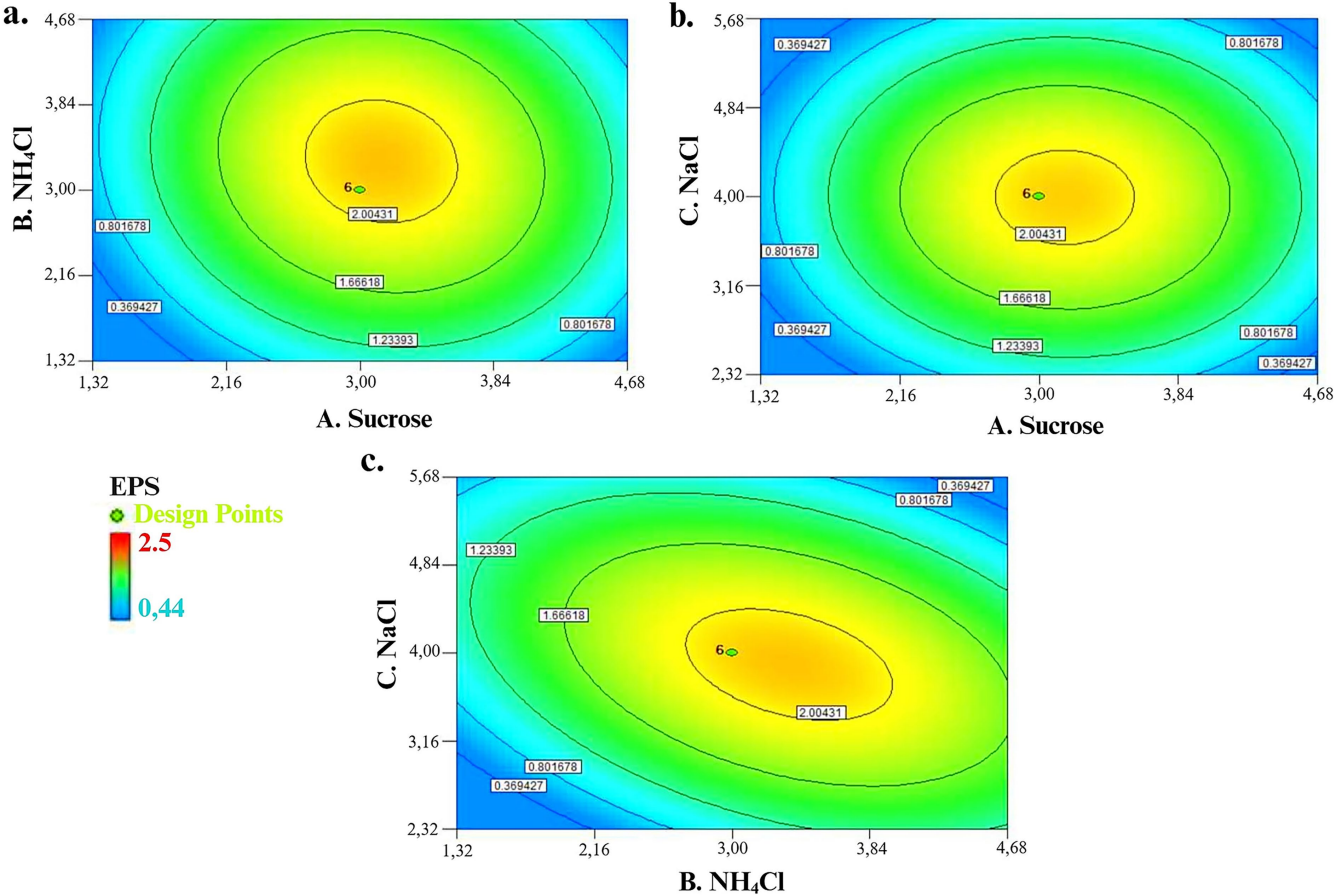
**(a)** Interaction effect of sucrose and NH_4_Cl on EPS production. **(b)** Interaction effect of sucrose and NaCl on EPS production. **(c)** Interaction effect of NH_4_Cl and NaCl on EPS production.

Contour plots (2-D) were a graphical representation to visualize the current state of the regression optimization model and to deduce the relationship between parameters and response. Circular or elliptical curves indicate that corresponding parameters have not interacted, while curves in the shape of hyperbolic and plateau refer to interaction. This study explained the relation between independent parameters and EPS production by 2-D contour plots response surface while others were kept at zero level (coded value). In Figure 5a, the interaction effect of 3% sucrose as carbon source and 3% NH_4_Cl as nitrogen source showed a maximum production of 1.97 g L^−1^ EPS, whereas minimum EPS production of 0.36 g L^−1^ was observed at minimum level for each carbon and nitrogen source concentrations. The maximum level of carbon and nitrogen sources also recorded a minimum EPS production of 0.8 g L^−1^. The interaction effect of sucrose and mineral salt NaCl on EPS production is explained in Figure 5b. Briefly, 3% of sucrose and 3% of NaCl resulted in 1.97 g L^−1^ of EPS production. Figure 5c revealed the interaction effect of NH_4_Cl and NaCl on EPS production; a high amount of EPS production (1.97 g L^−1^) was investigated when NH_4_Cl concentration was at 3% and NaCl concentration was at 4%. Minimum EPS production at low concentrations of NH_4_Cl and NaCl, but high concentrations of NaCl also revealed minimum EPS production.

### 3.5 Modeling of EPS production by artificial neural network prediction

**Figure 6:**
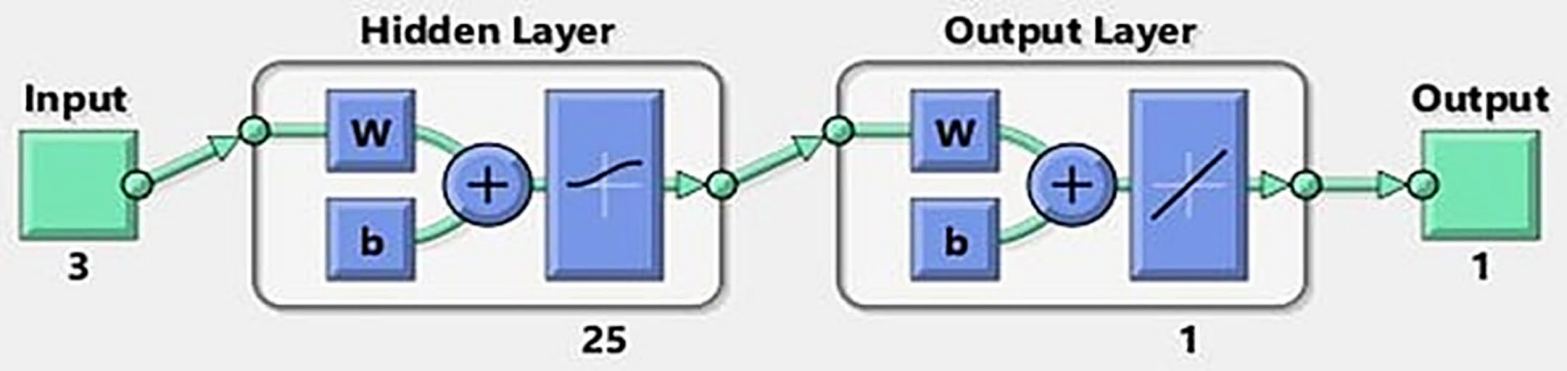
The obtained best ANN design.

As shown in Figure 6, an optimized artificial neural network (ANN) design with a 3–25-1 architecture was established. This model was employed to predict the production of EPS, where the network consists of three neurons in the input layer, a single hidden layer composed of 25 neurons, and one neuron in the output layer. The selection of this configuration was based on an optimization process aimed at maximizing the model’s accuracy in estimating EPS production, while ensuring a balance between learning capacity and generalization.

**Figure 7:**
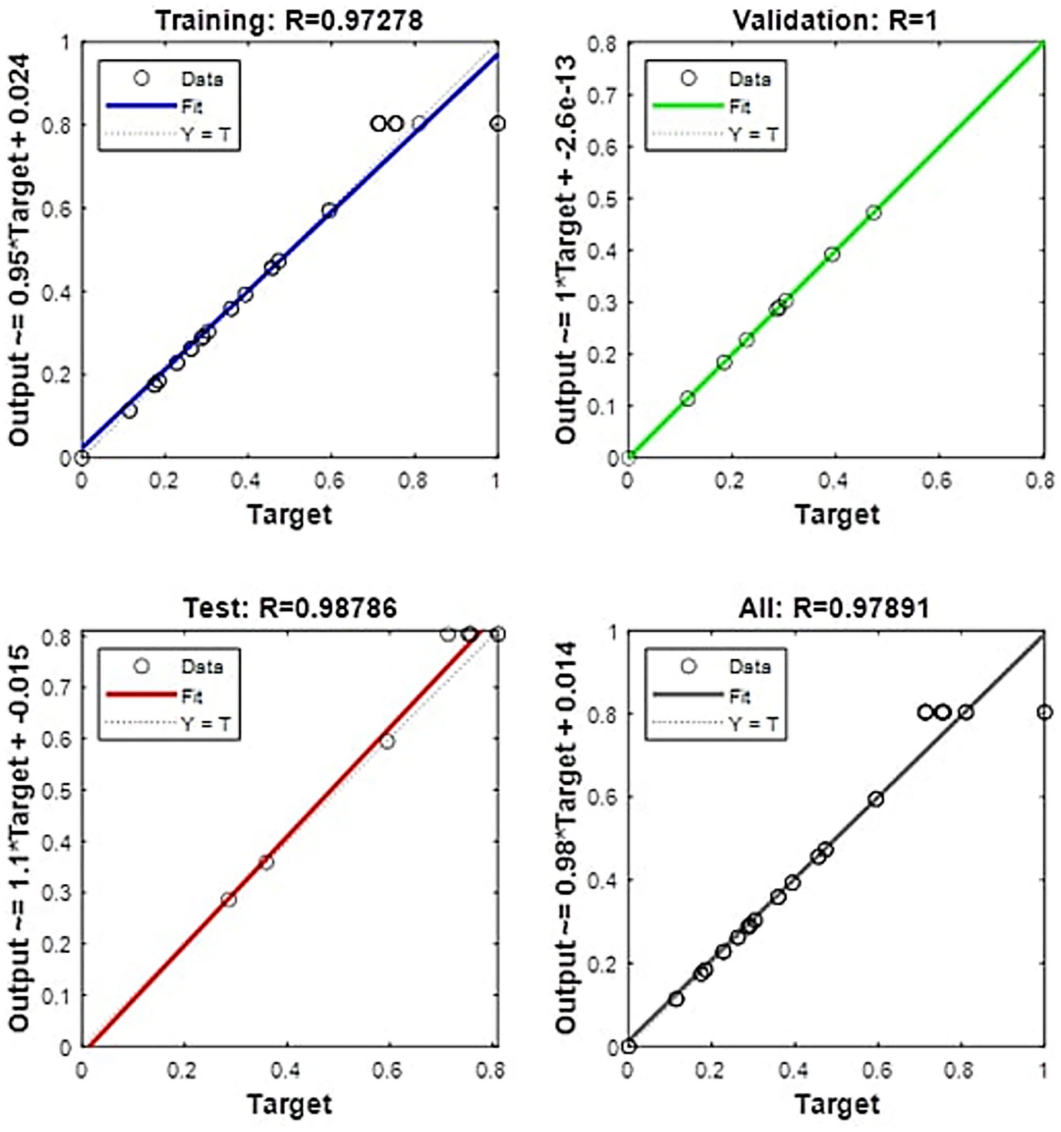
Linear fit of experimental and predicted EPS production using ANN.

In this study, a feedforward neural network (FFNN) was employed as a modeling tool to evaluate the impact of various factors on EPS production and to conduct kinetic experiments. The FFNN enabled the analysis of complex patterns in the experimental data and accurately predicted the influence of the studied variables. The model’s performance was assessed using two key metrics: relative error (RE) and the coefficient of determination (R^2^). The results showed a maximum RE of −10.9%, indicating a low deviation from the experimental values. Furthermore, the R^2^ value reached 0.9909 (Supplementary Figure 1), reflecting an excellent fit between the modeled and experimentally observed data. The results in Figure 7 demonstrate the model’s high accuracy, as the R^2^ values are close to 1 across all phases of the process (training, validation, and testing). Specifically, during the testing phase, the model achieved an R^2^ of 0.99626, indicating excellent predictive performance with previously unseen data. Similarly, in the training phase, the R^2^ value was 0.99248, and when considering all phases combined, an R^2^ of 0.99281 was obtained. These values confirm the robustness of the model in predicting EPS production and suggest that the ANN used possesses a suitable generalization capacity. The alignment of data points along the Y = T line in the regression plots further validates the model’s reliability, demonstrating a high level of agreement between predicted and experimental values.

### 3.6 Functional property elucidation of the EPS

#### 3.6.1 *In vitro* antioxidant and anticancer activity determination

**Figure 8:**
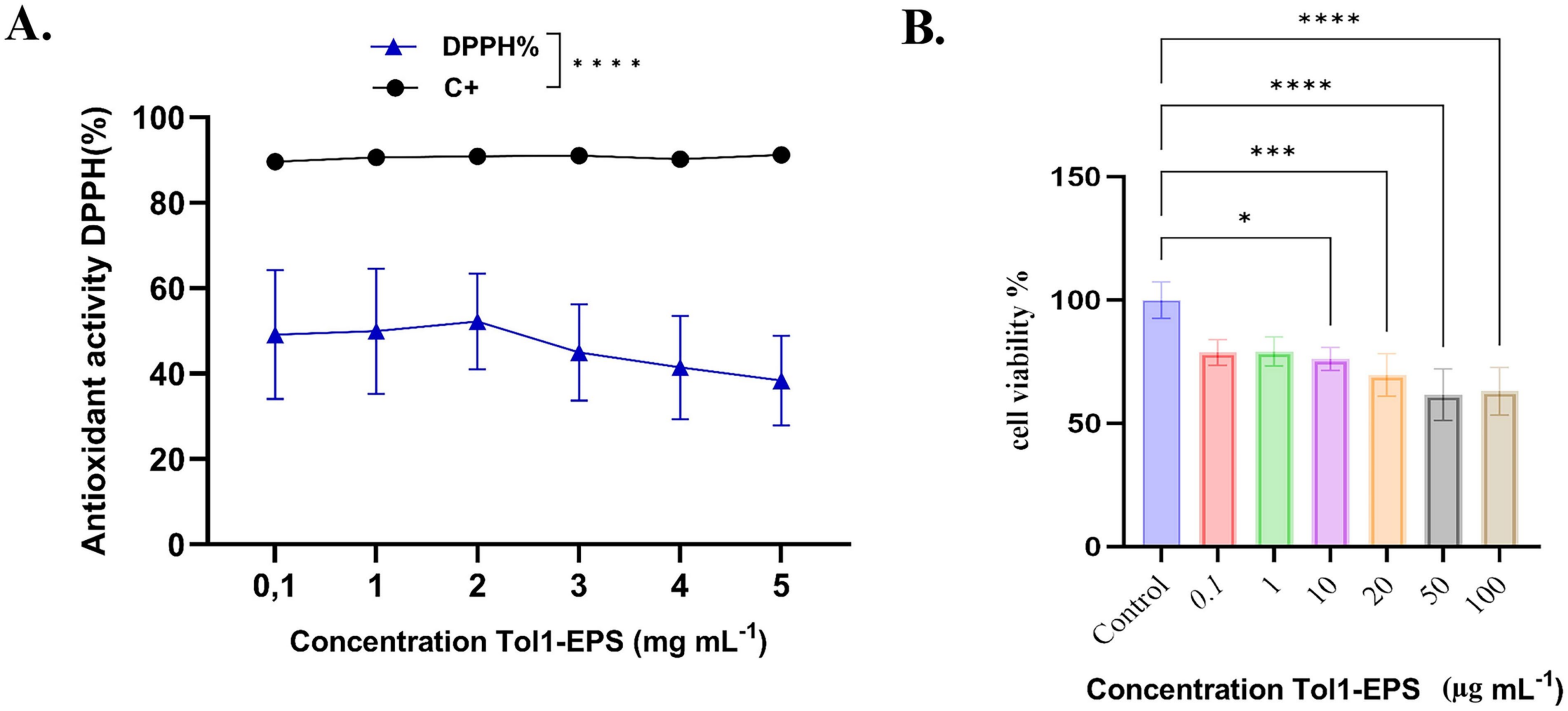
**(A)** Antioxidant activity (DPPH%) of the EPS produced by *B. licheniformis* Tol1. **(B)** Cell viability of AGS cells exposed to EPS produced by *B. licheniformis* Tol 1. Cell viability was assessed using the standard MTT assay. The Kruskal-Wallis test, followed by Dunn’s *post hoc* test, was used to compare the groups. Values of *p* ≤ 0.05 were considered statistically significant. *p ≤ 0.05, ***p < 0.001 and ****p < 0.0001. Data were expressed as mean ± SD of biological replicates.

Antioxidant activity was evaluated using the DPPH assay at increasing concentrations of Tol 1 EPS (0.1 to 5 mg mL^−1^). The results in Figure 8A show that EPS exhibits a moderate antioxidant capacity, with values ranging from ~45 to 60% DPPH radical inhibition, although with no clear upward trend with concentration. A slight decrease in antioxidant activity is observed at concentrations above 2 mg mL^−1^. The positive control (C^+^) maintains an activity close to 100%, indicating a statistically significant difference with respect to EPS (****, *p* < 0.0001). These results suggest that the EPS from *B. licheniformis* possesses antioxidant properties, although lower than those of the positive standard, and suggest a possible use as a moderate natural antioxidant agent.

#### 3.6.2 EPS produced by *B. licheniformis* Tol1 reduces gastric cancer cell viability

Cancer represents an urgent challenge for medicine, with gastric cancer being the fourth leading cause of cancer-related death worldwide and the leading cause of cancer mortality among men in Chile (Bray et al., 2024). In this context, EPS has emerged as a promising therapeutic solution due to its bioactive properties, including the ability to induce apoptosis and cell cycle arrest in tumor cells (Wu et al., 2021). In this study, a significant reduction in cell viability was observed in AGS cells from gastric cancer treated with 10, 20, 50, and 100 μg μL^−1^ compared to the untreated control group (Figure 8B). The most significant effects on cell viability were observed at concentrations of 50 and 100 μg μL^−1^, where it reached 61.62 and 63%, respectively, compared to the untreated control (set at 100%), reflecting reductions of 38.38 and 37%, respectively (*p* < 0.0001).

#### 3.6.3 Analysis of emulsification activity

**Figure 9:**
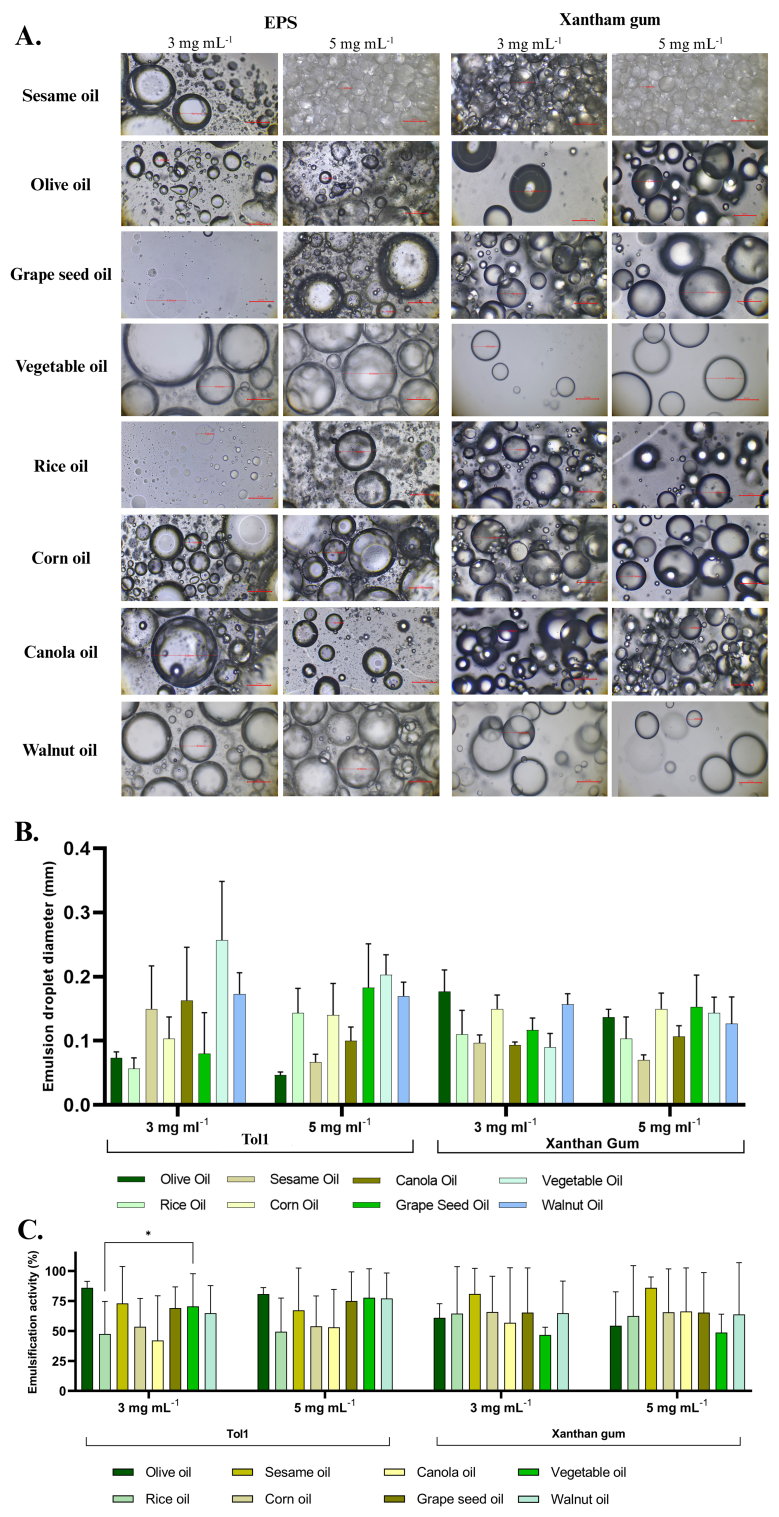
**(A)** Optical micrographs of oil–water emulsions stabilized with EPS of *B. licheniformis* Tol1 and Xanthan gum, **(B)** Emulsion droplet diameters against various food grade oils. **(C)** Emulsifying activity (%) of EPS produced by *B. licheniformis* Tol1 against different vegetable oils.

The emulsification activity of the EPS produced by *B. licheniformis* Tol1 was evaluated against various edible vegetable oils, including walnut, vegetable, grape seed, canola, corn, sesame, rice and olive oils, with xanthan gum as a commercial control (Figure 9). The emulsification index (E24) was measured at the initial time (0 h) and after 24 h, using EPS concentrations of 3 mg mL^−1^ and 5 mg mL^−1^. At the initial time (0 h), EPS exhibited strong emulsification across all tested oils, with the highest activities observed in grape seed oil and olive oil with emulsion values of (81.58% at 3 mg mL^−1^, and 92.12% at 5 mg mL^−1^) and (89.74% at 3 mg mL^−1^ and 84.62% at 5 mg mL^−1^) respectively, comparable to xanthan gum, which showed emulsification values ranging from 81.58 to 96.00% across different oils and concentrations. After 24 h, despite the expected natural phase separation, the study EPS retained notable stability, particularly at 5 mg mL^−1^, with the highest emulsification observed in walnut oil (62.16%), followed by grape seed (56.68%) and vegetable oils (55.26%). Xanthan gum exhibited slightly higher stability, confirming its established role as a long-term emulsifier. EPS-stabilized emulsions showed notable differences in droplet morphology and distribution depending on the oil type and EPS concentration (Figure 9A). At 3 mg mL^−1^, smaller droplets with a more homogeneous distribution were observed in oils such as olive, canola, and sesame, indicating efficient emulsification. At 5 mg mL^−1^, an increase in droplet size and coalescence was evident in some cases (e.g., sesame oil), suggesting potential supersaturation of the system. In comparison, xanthan gum-stabilized emulsions exhibited larger droplets and less dispersion in most oils, with a tendency to form less compact structures, especially in oils such as vegetable and rice oil. These results support the ability of Tol 1 EPS to stabilize fine and homogeneous emulsions, with comparable or superior performance to commercial emulsifiers, depending on the oil type. The emulsion droplet diameters of EPS from *B. licheniformis* Tol1 varied depending on the oil type and EPS concentration. At 3 mg mL^−1^, rice oil and olive oil showed the smallest droplet diameter (~0.07 mm), while vegetable and walnut oils exhibited the largest droplets (~0.30 mm) (Figure 9B). At 5 mg mL^−1^, a general reduction in droplet size was observed for most oils, indicating improved emulsification with higher EPS concentration. Overall, *B. licheniformis* Tol1 EPS demonstrated strong emulsification capacity, particularly at higher concentrations (Figure 9C). Compared to commercial bacterial EPS xanthan gum, Tol1 EPS maintained similar or smaller droplet sizes across most oils, highlighting its potential as an effective natural emulsifier.

## 4 Discussion

The exploration of thermophilic microorganisms from geothermal environments offers a promising avenue for the discovery of novel bioactive compounds, particularly microbial EPS, which exhibit multifunctional properties relevant to food, pharmaceutical, and environmental sectors (Upadhyaya et al., 2025). Despite significant progress in extremophile research, studies integrating genomic data with EPS functional and production analyses remain limited, especially in geothermal niches of South America, particularly Chile even being a natural laboratory to study extremophiles (Banerjee et al., 2022, 2024; Narsing Rao et al., 2024; Sarkar et al., 2024). Our study addresses this gap by characterizing a thermotolerant EPS-producing strain, *B. licheniformis* Tol1, isolated from the underexplored Tolhuaca hot spring in Chile. Beyond its EPS-producing capacity, *B. licheniformis* is widely recognized as a versatile microbial cell factory, extensively used in industrial biotechnology. It’s GRAS status, robust protein secretion system, and genetic tractability have facilitated its application in the large-scale production of enzymes, biopolymers, and other high-value metabolites (Muras et al., 2021; He et al., 2023). The adaptability of *B. licheniformis* to various environmental and nutritional conditions, alongside emerging synthetic biology tools, has further enhanced its utility in sustainable bioprocesses aligned with the principles of the circular bioeconomy. *B. licheniformis* is one of the most ubiquitous bacterial species, isolated from a remarkably diverse range of matrices, including marine sponges (Sayem et al., 2011), seaweed (Singh et al., 2011), salt flats (Insulkar et al., 2018), shallow marine hot springs (Arena et al., 2006), fermented medicine (Vinothkanna et al., 2021), soils (Liu et al., 2010), plant peel (Asgher et al., 2020), and even terrestrial geothermal ecosystems (Lakra and Sharma, 2024). Its broad ecological distribution underlines its metabolic versatility and adaptive capability at various growth conditions, making it a potent microbial cell factory for the sustainable production of bioactive EPSs with industrial potential.

The genome of *B. licheniformis* Tol1 revealed extensive metabolic versatility and stress adaptation potential, supported by 333 functional subsystems annotated via RAST. Genes associated with stress response, sporulation, oxidative stress protection, osmoregulation, and DNA repair were detected, consistent with its thermotolerant phenotype. Interestingly, the presence of antibiotic resistance genes and CRISPR-Cas locus in the Tol1 genome may also contribute to thermal stress resilience. Recent studies showed CRISPR-associated genes are upregulated in thermophiles like *Thermus thermophilus* and are implicated in broader stress adaptations (Karimi-Fard et al., 2025). Also, heat stress and antibiotic resistance share overlapping cellular targets and protective responses, including DNA repair and protein homeostasis mechanisms, suggesting that resistance determinants may offer cross-protection under high-temperature conditions as well (Rodríguez-Verdugo et al., 2020). These findings emphasize the critical role of prophages in shaping the genome and evolutionary trajectory of thermophilic *Bacillus* species, with implications for biotechnological applications and environmental resilience. Comprehensive genomic analysis also highlights the organism’s capability to survive in the geothermal environment, with significant prospects for industrial and scientific applications. Notably, we identified tyrosine kinase-related genes (*EpsD* and EpsC), which are implicated in EPS biosynthesis and biofilm development. The presence of *EpsD* and *EpsC* genes—encoding a tyrosine-protein kinase and its modulator, respectively—suggests a regulatory mechanism for EPS assembly and export, consistent with their roles in Wzy-dependent EPS biosynthetic pathways (Schmid, 2018). These genes have been implicated in polysaccharide co-polymerization and chain-length modulation in other *Bacillus* strains (Arbour et al., 2023). Additionally, several genes involved in glycosyltransferase activity, sugar nucleotide biosynthesis (e.g., *ugd*, *galE*), and polysaccharide transporter systems were identified in the annotated RAST subsystems, supporting the strain’s genomic potential for complex EPS synthesis. The association of EPS biosynthesis genes with biofilm matrix proteins such as *TasA* further underscores the link between EPS production and surface adherence, particularly under environmental stress (Okshevsky et al., 2018). Collectively, the presence of this EPS gene cluster, along with accessory genes for stress adaptation and DNA repair, suggests a tightly regulated EPS production network in Tol1, contributing to its ecological success in high-temperature geothermal ecosystems. These genomic signatures aligned with phenotypic traits such as robust biofilm formation and stable EPS production under moderately high temperatures (45–55°C). This echoes previous findings where EPS production by *Bacillus* strains was enhanced under osmotic or thermal stress, often mediated by these genetic pathways (Dogan et al., 2015; Narsing Rao et al., 2024). Optimization of EPS production was achieved through a multi-step strategy. OFAT analysis identified sucrose, NH_4_Cl, and NaCl as optimal nutrient components. RSM optimization using central composite design (CCD) further refined the medium composition, achieving a maximum yield of 2.5 g L^−1^. The model showed high significance (R^2^ = 0.934), consistent with findings by Premnath et al. (2021), where EPS production was influenced significantly by carbon, nitrogen and salt sources. Complementarily, ANN modeling using a multilayer perceptron (3–25-1) yielded a prediction accuracy of R^2^ = 0.9909, demonstrating superior predictive capability compared to RSM alone. Similar hybrid modeling approaches were employed by Mukherjee et al. (2019) and Suryawanshi et al. (2019), reinforcing the role of AI-driven process modeling in fermentation biotechnology. Actually, in order to strengthen the reliability of our EPS production model and validate the trends obtained through statistical optimization, we incorporated an ANN approach in addition to the traditional OFAT and RSM methods. While the OFAT method allowed preliminary screening to determine the most influential components (sucrose, NH₄Cl, and NaCl), which could not be efficiently analyzed via RSM due to its requirement for preselected variables. RSM, in turn, enabled us to examine interactions among these variables and establish an optimized condition, yielding a maximum EPS production of 2.11 g L^−1^ with high model fit. However, RSM predictions are limited by the assumption of quadratic behavior in the response surface. To overcome this and improve the predictive robustness, our feedforward ANN model achieved a prediction accuracy of R^2^ = 0.9909. The ANN did not surpass the experimental yield obtained from RSM but demonstrated superior generalization capacity across the input range, confirming the model’s reliability. This integrative approach—OFAT for variable selection, RSM for statistical optimization, and ANN for high-fidelity prediction—has been similarly adopted in EPS production studies (Suryawanshi et al., 2019) and reflects the increasing application of hybrid modeling in microbial process engineering.

The antioxidant potential of the Tol1-derived EPS, as revealed by DPPH-mediated radical scavenging assays, supports its role as a bioactive antioxidant polymer. This activity has been attributed to structural features such as uronic acid content, sulfation, and acetylation in *B. licheformis* produced EPS, as discussed in studies by Singh et al. (2011), and Vinothkanna et al. (2021). The EPS produced by Tol 1 demonstrated strong emulsifying capabilities across diverse edible oils, retaining up to 62.16% emulsion stability at 5 mg mL^−1^ after 24 h, particularly with walnut, grape seed, and vegetable oils. Although slightly lower than xanthan gum under prolonged incubation, the EPS displayed similar initial emulsification indices, indicating its potential as a natural food-grade emulsifier. Similar emulsification behavior has been reported in EPS from *B. licheniformis* and *B. coagulans* too (Kodali et al., 2009; Enrique et al., 2024), although our isolate maintained its stability with higher resilience at elevated incubation temperatures. Further, the anticancer potential of the EPS was evaluated against AGS gastric adenocarcinoma cells using MTT viability assays. The EPS induced a significant, dose-dependent reduction in cell viability, with the most pronounced cytotoxic effects observed at concentrations of 50 μg μL^−1^ and 100 μg μL^−1^, reducing cell viability by 38.38 and 37%, respectively. These results align with previous findings in which EPS from *Lactobacillus helveticus* (Li et al., 2014) and *Lactiplantibacillus plantarum* (Zhang et al., 2023) demonstrated potent anticancer effects via induction of apoptosis and cell cycle arrest. In another recent study on thermophilic bacterial EPS, the EPS produced by *Anoxybaciullus gonensis* showed significant anticancerous potential against YK-25A-549, DU-145, SHSY-5Y, and HT-29 cancer cells even at a concentration as low as 2.5 mg mL^−1^ (Karadayi et al., 2021). To date, no studies have been found evaluating the anticancer activity of EPS produced by *B. licheniformis* in gastric cancer. However, the anticancer potential of EPS derived from other bacteria has been investigated. Li et al. (2014) reported that both the purified EPS fractions (LHEPS-1, LHEPS-2, and LHEPS-3) and crude LHEPS from *Lactobacillus helveticus* MB2-1 reduced the proliferative capacity of human gastric cancer BGC-823 cells. Zhang et al. (2023) evaluated the effect of EPS from *Lactiplantibacillus plantarum* YT013 on the viability of AGS cells, demonstrating that EPS treatment significantly reduced cell viability in a dose-dependent manner. These results correlate with those obtained in the present study, further supporting the cytotoxic potential of EPS in gastric cancer. Cancer represents an urgent challenge for medicine, with gastric cancer being the fourth leading cause of cancer-related death worldwide and the leading cause of cancer mortality among men in Chile (Bray et al., 2024). In this context, EPS has emerged as a promising therapeutic solution due to its bioactive properties, including the ability to induce apoptosis and cell cycle arrest in tumor cells (Wu et al., 2021).

Our study, therefore, offers an integrative view, beginning from the genome and culminating in functional and bioactive EPS characterization to optimization of the bioprocess with AI-driven tools, highlighting the remarkable potential of thermophilic *Bacillus* strains from less explored Chilean geothermal sites. This not only broadens the global biogeography of industrially relevant thermophiles but also contributes novel genetic and functional insights to the field of microbial biotechnology. Importantly, our findings align with the goals of SDG 9 (Industry, Innovation, and Infrastructure) by promoting biotechnological innovation based on sustainable microbial resources and SDG 2 (Zero Hunger) by proposing a natural biopolymer with emulsifying and antioxidative properties suitable for food stabilization and nutritional enhancement.

## 5 Conclusion

In this study, *B. licheniformis* Tol1, a thermotolerant bacterium isolated from the Tolhuaca hot spring in Chile, was genomically and functionally characterized to explore its biotechnological potential. The strain demonstrated optimal growth at 55°C, producing EPS with notable biological properties. Genomic analysis revealed a 4.25 Mbp genome with 4,684 coding sequences, including genes associated with EPS biosynthesis (e.g., *epsD* and *epsC*), oxidative stress tolerance, and metal resistance. No virulence genes were identified, confirming their safety for industrial applications. The purified EPS showed strong emulsifying activity, reaching 92.12% with grape seed oil at 5 mg mL^−1^, and retained 62.16% stability after 24 h with walnut oil, performing comparably to commercial xanthan gum. Antioxidant assays indicated over 60% DPPH scavenging at 2 mg mL^−1^, while anticancer testing revealed that EPS significantly reduced AGS gastric cancer cell viability by 38.38 and 37% at concentrations of 50 and 100 μg μL^−1^, respectively (*p* < 0.0001). Additionally, EPS production was optimized to 2.11 g L^−1^ using response surface methodology and validated by an ANN model (R^2^ = 0.99), supporting its scalability. These results demonstrate that *B. licheniformis* Tol1 is a robust candidate for developing sustainable bioproducts. Its EPS holds promise as a natural emulsifier and antioxidant additive in functional foods, as well as a potential bioactive compound for cancer prevention strategies. The integration of genomics, ANN-based prediction, and bioactivity assays supports the development of eco-friendly biotechnological platforms, while promoting responsible use of Chile’s geothermal microbial resources. This work underscores the critical value of extremophiles in advancing next-generation, circular bioeconomy solutions and the conservation of natural resources.

## Data Availability

The original contributions presented in the study are publicly available. This data can be found here: https://www.ncbi.nlm.nih.gov/, JBNNEF000000000.
