# Supplementary material for: Genomic basis and functional characterization of the exopolysaccharide production by a thermotolerant Bacillus isolated from Tolhuaca hot spring
Source: Front Microbiol. 2025 Aug 4;16:1622325. doi: 10.3389/fmicb.2025.1622325 (PMC12359474; doi:10.3389/fmicb.2025.1622325)
Supplement: Supplementary file 1 [file Table_1.DOCX]

**Table S1.** One Factor at a Time Assessment (OFAT)

| **Factors** | **Rep 1** | **Rep 2** | **Rep 3** | **Rep 4** |
| --- | --- | --- | --- | --- |
| **50°C** | ++ | ++ | ++ | ++ |
| **55°C** | +++ | +++ | +++ | +++ |
| **60°C** | +++ | +++ | +++ | +++ |
| **65°C** | +++ | +++ | +++ | +++ |
| **70°C** | ++ | ++ | ++ | ++ |
| **2% NaCl** | +++ | +++ | +++ | +++ |
| **5% NaCl** | +++ | +++ | +++ | +++ |
| **7% NaCl** | ++ | ++ | ++ | ++ |
| **10% NaCl** | + | + | + | + |
| **pH 5** | 0.191 | 0.195 | 0.269 | 0.205 |
| **pH 6** | 0.125 | 0.128 | 0.161 | 0.146 |
| **pH 7** | 0.153 | 0.178 | 0.192 | 0.165 |
| **pH 8** | 0.184 | 0.169 | 0.211 | 0.195 |
| **Glucose** | +++ | +++ | +++ | +++ |
| **Sucrose** | +++ | +++ | +++ | +++ |
| **Galactose** | +++ | +++ | +++ | +++ |
| **Mannose** | +++ | +++ | +++ | +++ |
| **Yeast extract** | ++ | ++ | ++ | ++ |
| **Meat extract** | ++ | ++ | ++ | ++ |
| **Peptone** | ++ | ++ | ++ | ++ |
| **NH_4_Cl** | +++ | +++ | +++ | +++ |

(+++) High growth and viscosity, (++) Moderate growth and viscosity, (+) Low growth and viscosity

**Table S2:** Central Composite Design (CCD) for the production of EPS from *Bacillus licheniformis* TOL1.

| **Std** | **Run** | **Point type** | **Parameters** | | | **EPS g /L** | |
| --- | --- | --- | --- | --- | --- | --- | --- |
|  |  |  | **% Sucrose** | **% NH_4_Cl** | **% NaCl** | **Actual** | **Prediction** |
| 9 | 1 | Axial | 1.32 | 3 | 4 | 0.68 | 0.68 |
| 19 | 2 | Center | 3 | 3 | 4 | 1.91 | 2.07 |
| 2 | 3 | Fact | 4 | 2 | 3 | 0.82 | 0.84 |
| 16 | 4 | Center | 3 | 3 | 4 | 1.91 | 2.07 |
| 5 | 5 | Fact | 2 | 2 | 5 | 0.8 | 0.91 |
| 20 | 6 | Center | 3 | 3 | 4 | 2.11 | 2.07 |
| 11 | 7 | Axial | 3 | 1.32 | 4 | 1.18 | 1.06 |
| 10 | 8 | Axial | 4.68 | 3 | 4 | 1.03 | 1.12 |
| 14 | 9 | Axial | 3 | 3 | 5.68 | 1.04 | 1.02 |
| 15 | 10 | Center | 3 | 3 | 4 | 1.99 | 2.07 |
| 4 | 11 | Fact | 4 | 4 | 3 | 1.67 | 1.49 |
| 12 | 12 | Axial | 3 | 4.68 | 4 | 1.38 | 1.59 |
| 13 | 13 | Axial | 3 | 3 | 2.32 | 0.91 | 1.02 |
| 3 | 14 | Fact | 2 | 4 | 3 | 1.415 | 1.33 |
| 8 | 15 | Fact | 4 | 4 | 5 | 1.065 | 1.00 |
| 17 | 16 | Center | 3 | 3 | 4 | 2.50 | 2.07 |
| 1 | 17 | Fact | 2 | 2 | 3 | 0.44 | 0.44 |
| 6 | 18 | Fact | 4 | 2 | 5 | 1.25 | 1.27 |
| 18 | 19 | Center | 3 | 3 | 4 | 2.00 | 2.07 |
| 7 | 20 | Fact | 2 | 4 | 5 | 0.98 | 0.89 |


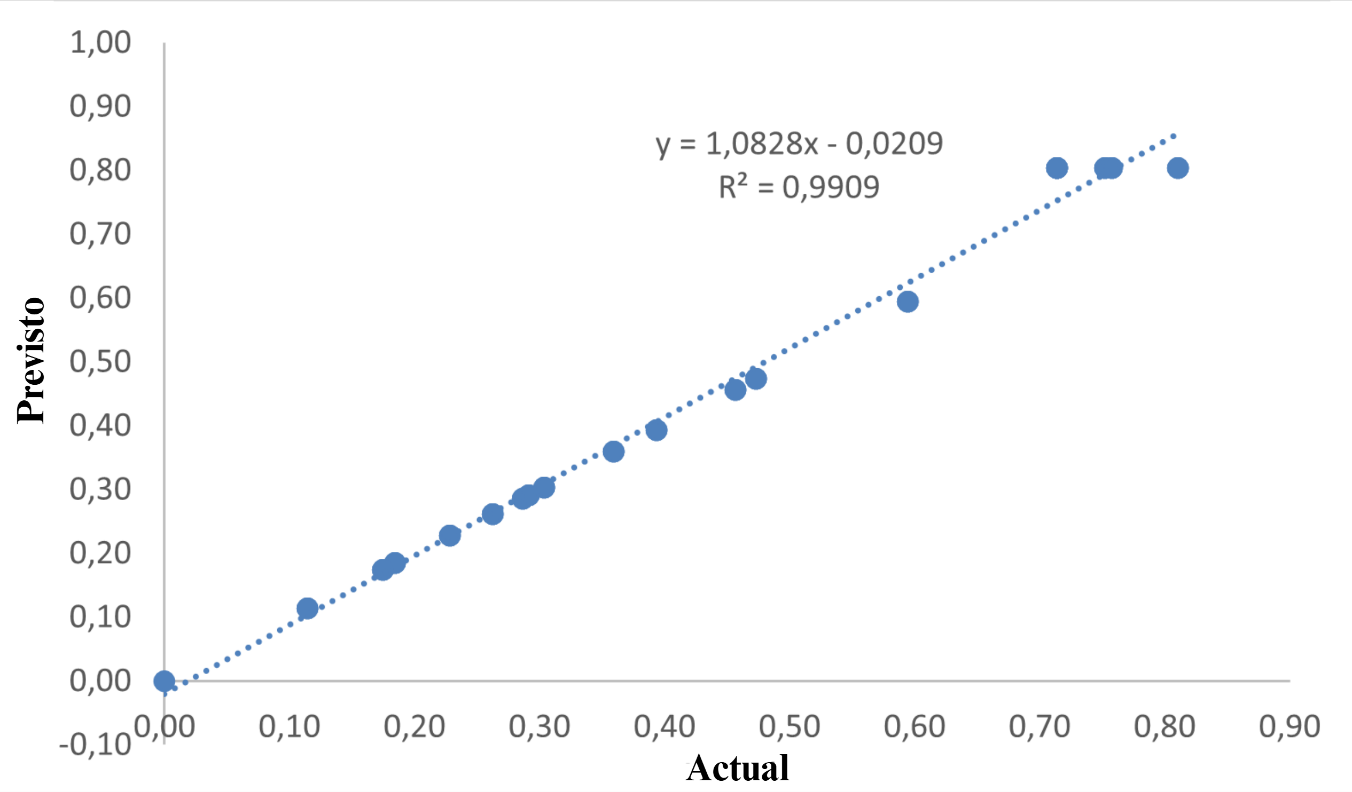


**Figure S1.** Coefficient of correlation (R^2^) of the neural network model.


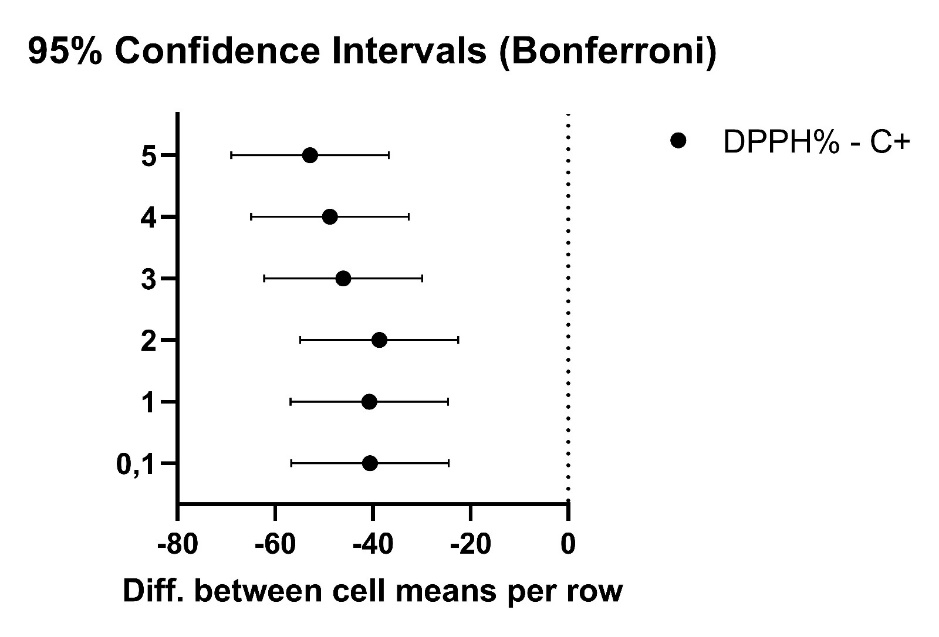


**Figure S2.** Statistical comparison of the antioxidant activity (DPPH%) of the EPS produced by Bacillus licheniformis Tol1 versus the positive control (C+) using 95% confidence intervals with Bonferroni correction.
